# Intensification of Treatment for Angiosarcoma of the Breast with Accelerated Hyperfractionated Radiation, Hyperthermia, and Surgical Resection

**DOI:** 10.7759/cureus.1406

**Published:** 2017-06-28

**Authors:** Jason K Molitoris, Arpit Chhabra, James W Snider, Nicole Harvilla, Nkechi Okonkwo, Elizabeth M Nichols, Zeljko Vujaskovic, Olga B Ioffe, Susan B Kesmodel, Steven J Feigenberg

**Affiliations:** 1 Department of Radiation Oncology, University of Maryland School of Medicine, Baltimore, USA; 2 Pathology, University of Maryland School of Medicine, Baltimore, USA; 3 Surgery, University of Maryland School of Medicine, Baltimore, USA

**Keywords:** angiosarcoma, hyperthermia, secondary malignancy, radiation

## Abstract

Angiosarcoma (AS) of the breast is a rare malignancy most commonly encountered as a secondary malignancy after the treatment of breast cancer with or without adjuvant radiation. The prognosis for secondary AS is poor, with reported five-year overall survival rates ranging from 10%-43%. The establishment of local control is vital to prognosis, yet patients often die with locally progressive disease. Multiple local therapies have been employed including surgery alone, surgery followed by radiation, and concurrent radiation and hyperthermia. Here, we report a case of secondary AS that occurred after breast conserving therapy and adjuvant radiation for ductal carcinoma in situ (DCIS). After initial surgical excision and subsequent local recurrence, our patient was treated with a novel treatment intensification strategy including neoadjuvant, accelerated hyperfractionated radiation with concurrent hyperthermia, followed by total mastectomy and flap reconstruction. The final pathologic evaluation demonstrated a near-complete response to induction thermoradiotherapy.

## Introduction

Secondary angiosarcoma (AS) of the breast is a rare malignancy (0.2% of all breast cancer patients) that can occur with or without adjuvant radiation, and typically has limited therapeutic options. It often presents with diffuse skin involvement and arises within or near the prior radiation field [1-3]. There is no proven effective chemotherapy regimen, and the majority of patients experience significant local morbidity. Single modality approaches typically result in poor rates of local control. Surgical resection, when possible, is the preferred initial strategy. However, surgery alone still results in local failure rates of 22%-92% and does depend on the extent of surgery; one series demonstrated an 80% and 50% recurrence after lumpectomy and mastectomy, respectively [3-5]. Results without surgery are poor, with one series demonstrating 91% persistent or recurrent disease [5]. Therefore, multiple efforts have been undertaken to improve local control by combining locoregional therapies.

Several methods have been examined to increase the efficacy of radiation therapy. Of these, one promising approach is the use of accelerated hyperfractionated radiation therapy, initially reported by Feigenberg, et al. and updated most recently in 2014, using radiation therapy twice daily (BID) and three times daily (TID) [6-7]. An alternative approach undertaken in the Netherlands is the use of external thermal therapy (ETT) concurrent with radiation [8]. Re-irradiation and concurrent ETT is an effective treatment for superficial tumors with randomized evidence suggesting improved complete response and local control [9]. Our clinic has experience with both ETT for recurrent breast cancers as well as accelerated hyperfractionated radiation for AS, and here, we describe a case utilizing both approaches prior to planned surgical resection.

## Case presentation

**Figure 1:**
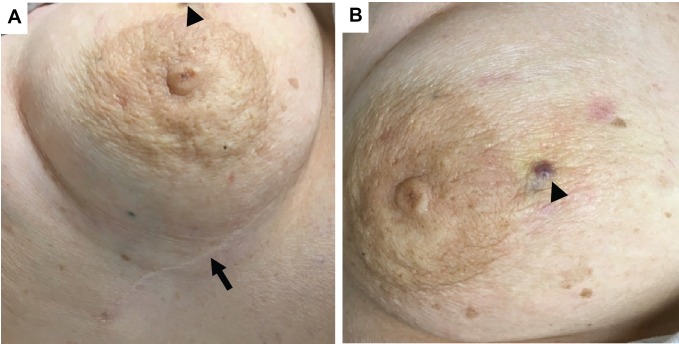
Angiosarcoma at recurrence Photographs of the right breast at the time of AS recurrence. A. Lateral right breast with a scar from the initial AS resection (arrow) and edge of violaceous recurrence (arrow head). B. Medial right breast with violaceous nodular recurrence prior to biopsy (arrow head).

**Figure 2:**
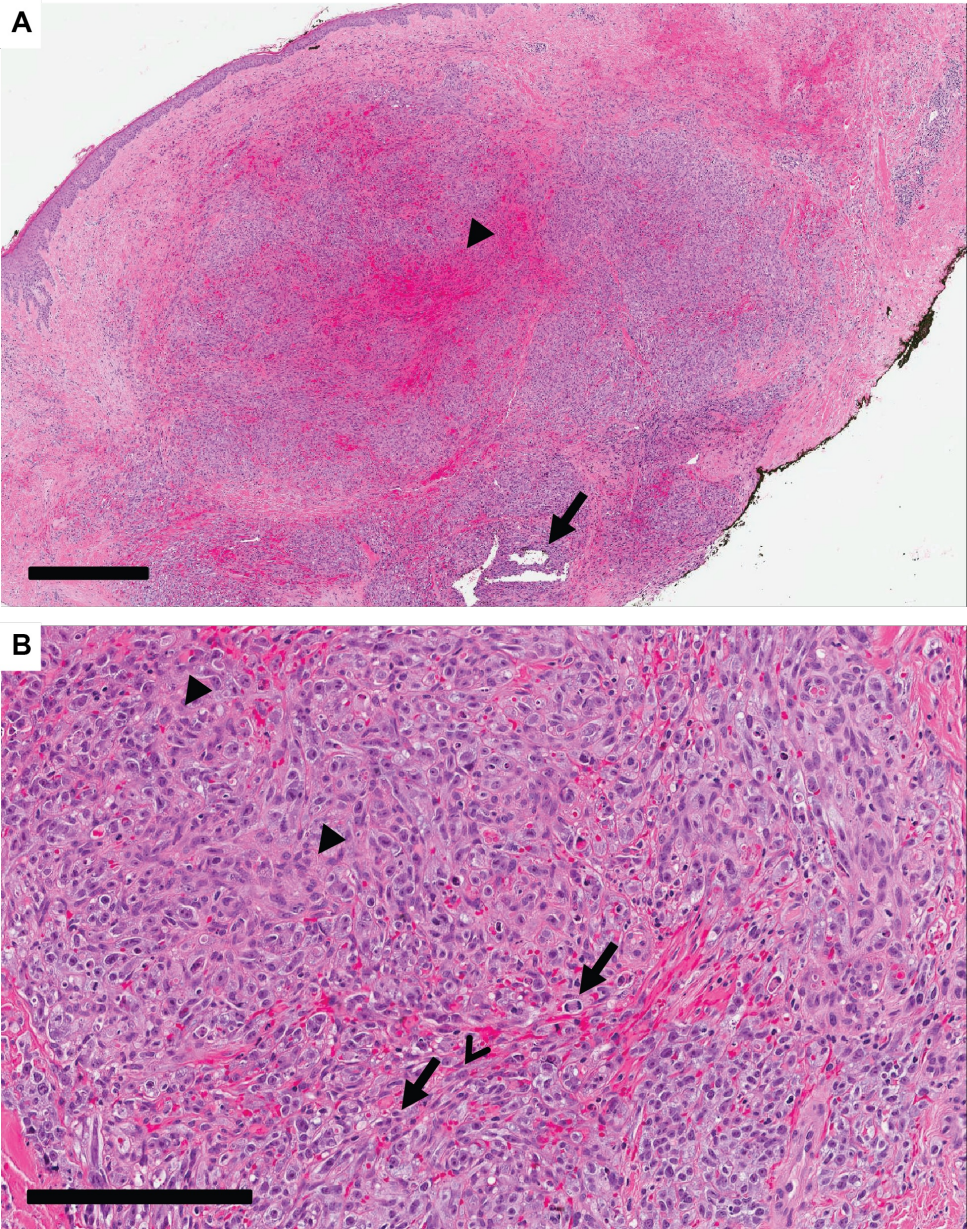
Biopsy micrographs Haemotoxylin and Eosin stained biopsy of AS with skin involvement. A. Low-magnification view demonstrating dilated vascular channels (arrow) and extravasated red blood cells forming blood lakes (arrow head) (black bar - 600µm). B. Higher power demonstrating atypical endothelial cells with hyperchromatic nuclei, pleomorphism, and mitosis (arrow). This field shows a solid growth pattern with epithelioid (arrow head) and slightly spindled cells (open arrow head) (black bar - 200µm).

A 72-year-old Caucasian female was initially treated with breast conserving surgery and adjuvant radiation (in 2005) to the right breast for ductal carcinoma in situ (DCIS). In the fall of 2014, she noted a new cutaneous lesion on the right lateral breast. She underwent a wide local excision in October 2014 which demonstrated AS; 2 cm in maximal dimension. She was recommended for close surveillance until, in late 2016, she noted a small violaceous area of skin thickening at the 3 o’clock position with associated induration. The residual scar from the initial resection was at least 10 cm from the new violaceous lesion (Figure 1A-1B). A biopsy performed in January 2017 demonstrated recurrent AS, moderately differentiated (Figure 2).

Her case was discussed in the multidisciplinary tumor board. Accelerated hyperfractionated radiation and concurrent ETT was recommended as neoadjuvant therapy to be followed by mastectomy and flap reconstruction. Pre-operative therapy was recommended as it would allow for removal of the majority of the re-irradiated tissue at the time of surgery. 

**Figure 3:**
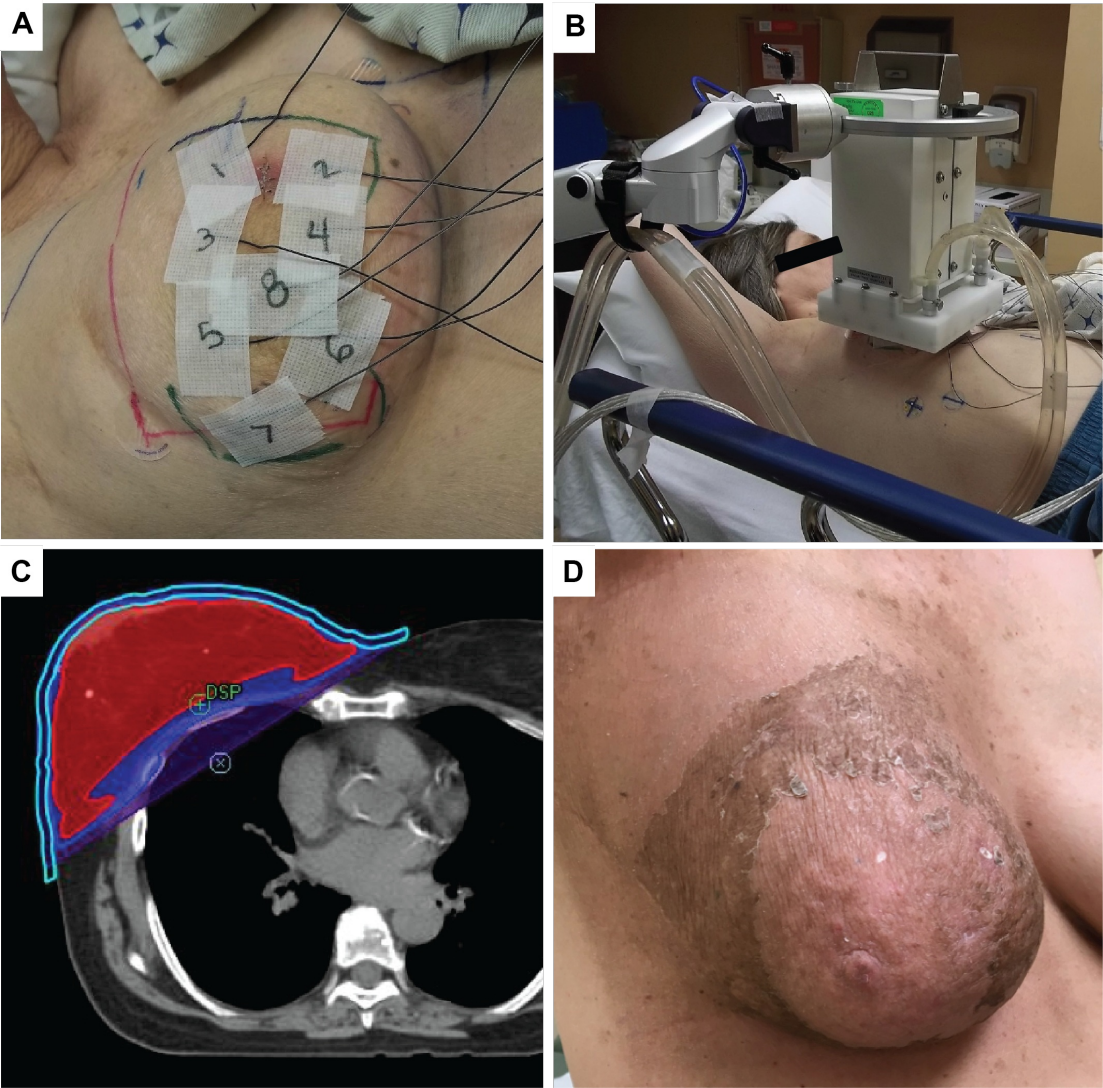
Neoadjuvant treatment Neoadjuvant accelerated radiation and concurrent hyperthermia. A. ETT superficial thermistors set on the patient prior to each ETT treatment. B. Medium ETT applicator in use. C. Axial CT with blous (light blue outline) and radiation isodose lines (red – 100%, dark blue – 95%, purple – 50%). D. Clinical response four weeks post treatment demonstrating the absence of the violaceous nodule and a resolution of moist desquamation over the treated area.

For ETT administration, the BSD-500 unit was used for all treatments. The surface temperature was monitored during each administration utilizing the incorporated thermistors of the device. Eight thermistors were placed across the breast to prevent unrecognized heterogeneity in thermal dose (Figure 3A). Twice weekly, ETT was administered within 30 minutes of radiation for a total of six treatments with a tumor target temperature of 40-43◦C, with a water bolus temperature of 40◦C. The ETT duration was 50-60 minutes (median, 60 minutes) per session, with the medium applicator (MA-100) in a single field technique (Figure 3B).

She underwent computed tomography simulation (CT-Sim) and was scheduled for twice-daily radiation using a 3D-conformal technique with tangent fields and 0.5 cm tissue equivalent bolus to encompass the lesion plus a 5 cm margin and all of the previously irradiated breast tissue (Figure 3C). She was prescribed 4450 centigrey (cGy), of which 4200cGy was delivered in 150cGy BID treatments and a single-daily 250cGy fraction due to dosing schedule issues within a day.

She tolerated radiation and concurrent ETT well without any treatment breaks. She did experience pain (requiring narcotic use) and moist desquamation confluent over the right breast which was managed conservatively with silvadene. She was evaluated four weeks later with significant improvement in her pain and skin (Figure 3D). Seven weeks post-thermoradiotherapy, she underwent a right total mastectomy with latissimus dorsi rotational flap and left breast reduction mammoplasty without perioperative complications.

**Figure 4:**
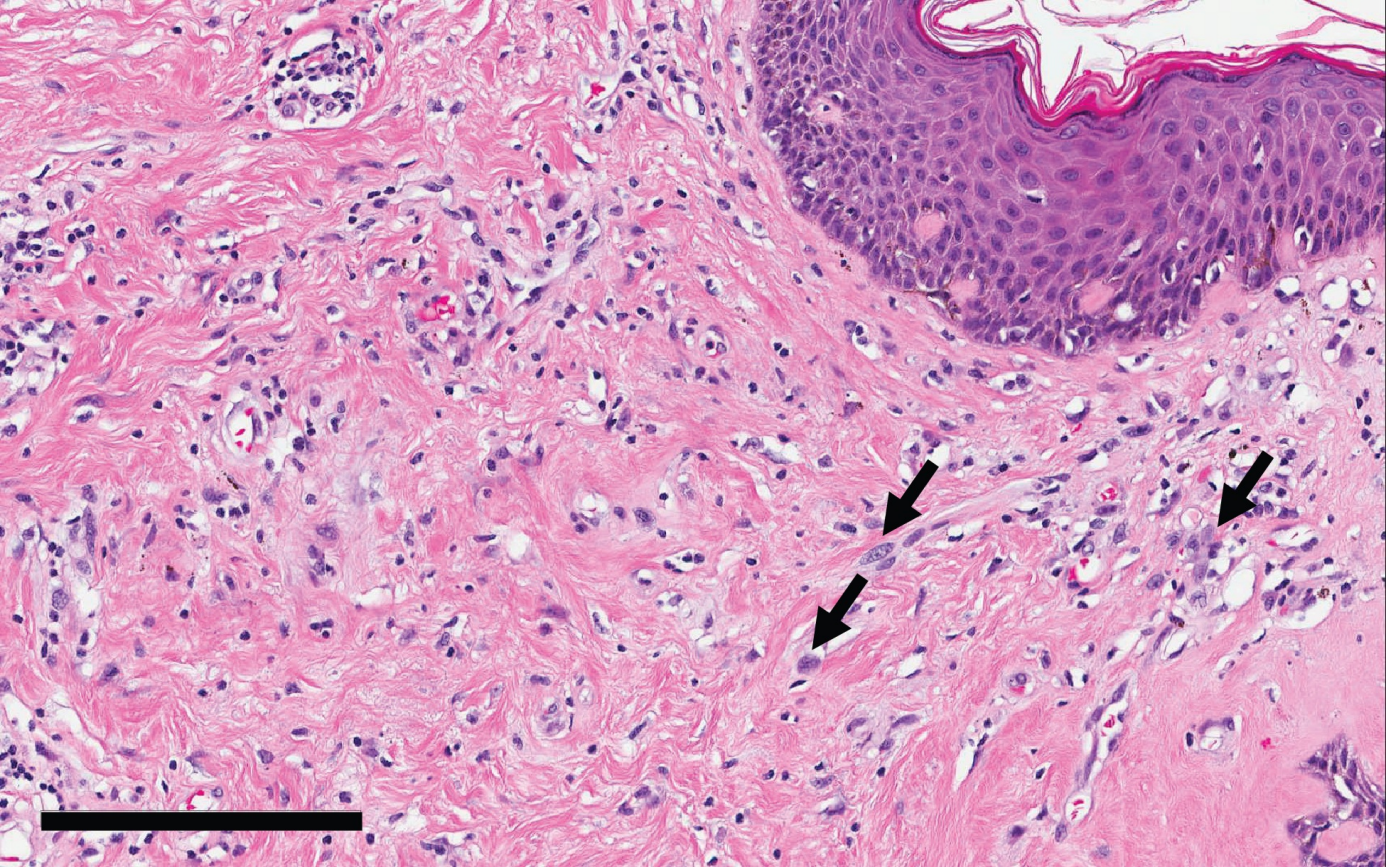
Mastectomy Micrograph Post resection focal residual AS at high power. Atypical endothelial cells and vascular channels infiltrate the connective tissue of the dermis (arrow)(black bar - 200µm).

Pathology demonstrated near pathological complete response with only a microscopic focus of residual cutaneous angiosarcoma (Figure 4). She healed well after surgery and is currently five months from diagnosis, and without any evidence of the disease. This research was performed on an institutional review board approved protocol for evaluation of the safety of thermal therapy with concurrent radiation.

## Discussion

AS of the breast is a rare soft tissue tumor of endothelial differentiation. This tumor accounts for 1% of all soft tissue tumors found in the breast and can present as a primary or secondary lesion. Primary lesions are typically idiopathic and are observed in women aged 30-50 years, presenting with a palpable mass. The lesion most often arises from the parenchymal blood vessels and can extend to involve the overlying skin. In contrast, secondary lesions typically present in older women. These patients often have a history of breast cancer treatment including adjuvant radiation or axillary lymph node dissection with subsequent development of longstanding lymphedema. Secondary AS can occur after surgery alone and has been described as Stewart-Treves syndrome when it occurs in the setting of chronic lymphedema, suggesting a multifactorial etiology.

AS are highly vascular tumors that are prone to excessive bleeding after a biopsy. Grossly, the lesions are firm, ill-defined, and violaceous with blue/purple nodules. The lesions have an average size of 5 cm and when there is skin involvement, patches of skin discoloration can be appreciated. Histologically, AS demonstrate dilated anastomosing vascular channels lined by plump endothelial cells; however, there is a morphologic spectrum of the lesion depending on the differentiation. Low-grade lesions exhibit an infiltrative pattern but otherwise appear benign with vascular channels lined by hyperchromatic nuclei with slight cytologic atypia. Intermediate grade lesions are similar but with increased mitotic activity, endothelial tufting, and foci of solid growth pattern. High-grade lesions demonstrate high mitotic activity, and they display marked cytologic atypia, necrosis, and solid growth with epithelioid and spindled cytology. The vascular origin of high-grade lesions can be difficult to identify; however, immunohistochemical staining with endothelial markers can aid in diagnosis. CD31, a membranous immunostain that outlines individual cells, especially at cell junctions, is the most sensitive and specific endothelial marker [10].

Herein, we present a case report of a woman diagnosed with a secondary AS after breast conservation therapy for DCIS. This AS recurred after wide local excision alone, a common occurrence in up to two-thirds of patients undergoing surgery alone [8]. Wide resection does appear to be helpful, as one series of nine patients had only two local failures with planned surgical margin of at least 3 cm, although this is not always possible [4].

While evidence for recommendations in this disease entity is limited, multi-modality therapy is generally favored when feasible. Altered fractionation and especially accelerated hyperfractionation radiation are used at the University of Florida to offer a radiobiologic advantage with promising results [6-7]. An initial report of three consecutive patients who developed angiosarcoma after breast conserving therapy demonstrated the relative safety and long-term disease control associated with BID or TID radiotherapy in the pre- or postoperative settings [6]. Recently, this experience has been updated with a total of 14 patients [7]. The favored fractionation course has been TID (n=13) with 1 Gy/fraction separated by at least 4 hours. Prescribed doses were 4500cGy to clinically uninvolved tissues 5-10 cm around clinically involved sites, 6000cGy to tissue within 2 cm of the primary tissue, and 7500cGy to gross disease (unresectable disease). This technique has yielded a 10-year progression-free survival rate of 64% with a cause-specific survival of 71%, representing particularly excellent results in this challenging, rare disease. In our case, after multidisciplinary tumor board discussion, we elected to employ neoadjuvant, accelerated hyperfractionated radiation before definitive resection.

An alternative mechanism used in superficial tumors to increase the effectiveness of radiation is the addition of concurrent hyperthermia. Concurrent hyperthermia is an effective adjunct to increase radiation sensitivity with randomized clinical data demonstrating promising results for superficial tumors [9]. ETT has been previously employed in AS and a report on its use in 23 patients with concurrent daily radiation demonstrated a complete response rate of 56%, and a three-year local control of 46% for those who also underwent surgery with late grade 4 toxicity in two patients [8]. We have noted modest toxicities with hyperthermia in our clinical practice. For our patient, we added concurrent ETT twice weekly during accelerated hyperfractionated radiation. As was expected, the patient did experience an acute grade 3 dermatitis reaction; however, she improved one month after treatment with conservative therapy and was able to undergo resection and flap reconstruction. Continued close monitoring will be required to evaluate for long-term treatment complications including fibrosis, joint stiffness, and pain.

Pathological evaluation of the resected specimen demonstrated significant treatment effect with only a microscopic focus of residual disease. After accelerated hyperfractionated radiation, the University of Florida has reported complete response in all seven patients who underwent subsequent surgical resection after a high-dose hyperfractionated radiation [7]. In the reported series combining radiation and hyperthermia, one of three patients undergoing a neoadjuvant strategy achieved a pathological complete response [8]. Given that our patient had an intermediate grade tumor, it is possible that with additional time between neoadjuvant therapy and surgery, a complete pathological response may have been appreciated.

## Conclusions

Secondary AS is a challenging disease entity for clinical management. Identifying optimal treatment strategies is also difficult given the rarity of the disease and heterogeneity of presentations. Recently, there have been significant efforts to improve local outcomes with combined therapies and treatment intensification. Here, we report, for the first time, the use of neoadjuvant, accelerated hyperfractionated radiation with concurrent hyperthermia followed by surgical resection for a secondary AS. Toxicities were modest with an excellent pathologic response and the promise of increased efficacy. Continued surveillance for tumor control and side effects in our patient is needed and accumulation of additional patients will determine if this is a viable treatment strategy for this rare disease.
